# Multi-Omics Approach Points to the Importance of Oxylipins Metabolism in Early-Stage Breast Cancer

**DOI:** 10.3390/cancers14082041

**Published:** 2022-04-18

**Authors:** Dmitry V. Chistyakov, Mariia V. Guryleva, Elena S. Stepanova, Lyubov M. Makarenkova, Elena V. Ptitsyna, Sergei V. Goriainov, Arina I. Nikolskaya, Alina A. Astakhova, Anna S. Klimenko, Olga A. Bezborodova, Elena A. Rasskazova, Olga G. Potanina, Rimma A. Abramovich, Elena R. Nemtsova, Marina G. Sergeeva

**Affiliations:** 1 Belozersky Institute of Physico-Chemical Biology, Lomonosov Moscow State University, 119992 Moscow, Russia; alina_astakhova@belozersky.msu.ru (A.A.A.); sergeeva@belozersky.msu.ru (M.G.S.); 2 Faculty of Bioengineering and Bioinformatics, Lomonosov Moscow State University, 119234 Moscow, Russia; maria.gur@fbb.msu.ru (M.V.G.); ptitsynaev@my.msu.ru (E.V.P.); ar-nikolya@fbb.msu.ru (A.I.N.); 3 Peoples’ Friendship University of Russia (RUDN University), 6 Miklukho-Maklaya Street, 117198 Moscow, Russia; stepanova_es@pfur.ru (E.S.S.); makarenkova-lm@rudn.ru (L.M.M.); goryainov_sv@rudn.university (S.V.G.); klimenko-as@rudn.ru (A.S.K.); 4 National Medical Radiology Research Center, P. Hertsen Research Institute of Oncology, 125284 Moscow, Russia; olgabezborodova@yandex.ru (O.A.B.); rasskaz2@yandex.ru (E.A.R.); nemtz@yandex.ru (E.R.N.); 5 Faculty of Medicine, Lomonosov Moscow State University, 119991 Moscow, Russia; ogpotanina@fbm.msu.ru (O.G.P.); raabramovich@fbm.msu.ru (R.A.A.)

**Keywords:** COX, CYP450, LOX, oxylipins, PUFAs, lipidomics, UPLC-MS/MS, breast cancer, transcriptomics, anandamide

## Abstract

**Simple Summary:**

On a system level, multi-omics approaches allow studying oxylipins, metabolites of omega-3 or omega-6 polyunsaturated fatty acids. We compared the ultra-high-performance liquid chromatography-mass spectrometry (UPLC-MS/MS) oxylipin profile signatures in the blood plasma of 152 healthy volunteers (HC) and 169 patients with different stages of breast cancer (BC). We identified 18 differentially expressed oxylipins between BC vs. HC patients, including anandamide, prostaglandins, and hydroxydocosahexaenoic acids. To integrate lipidomics, transcriptomics, and genomics data, we analyzed a transcriptome of 10 open database datasets obtained from tissues and blood cells of BC patients and SNP data for 33 genes related to oxylipin metabolism and revealed 19 changed genes, among them CYP2C19, PTGS2, HPGD, and FAAH included in the list of DEGs in the analysis of transcriptomes and the list of SNPs associated with BC. Our data allow us to suppose that oxylipin profiles can be used to evaluate the early stages of breast cancer.

**Abstract:**

The involvement of oxylipins, metabolites of polyunsaturated fatty acids, in cancer pathogenesis was known long ago, but only the development of the high-throughput methods get the opportunity to study oxylipins on a system level. The study aimed to elucidate alterations in oxylipin metabolism as characteristics of breast cancer patients. We compared the ultra-high-performance liquid chromatography-mass spectrometry (UPLC-MS/MS) oxylipin profile signatures in the blood plasma of 152 healthy volunteers (HC) and 169 patients with different stages of breast cancer (BC). To integrate lipidomics, transcriptomics, and genomics data, we analyzed a transcriptome of 10 open database datasets obtained from tissues and blood cells of BC patients and SNP data for 33 genes related to oxylipin metabolism. We identified 18 oxylipins, metabolites of omega-3 or omega-6 polyunsaturated fatty acids, that were differentially expressed between BCvsHC patients, including anandamide, prostaglandins and hydroxydocosahexaenoic acids. DEGs analysis of tissue and blood samples from BC patients revealed that 19 genes for oxylipin biosynthesis change their expression level, with CYP2C19, PTGS2, HPGD, and FAAH included in the list of DEGs in the analysis of transcriptomes and the list of SNPs associated with BC. Results allow us to suppose that oxylipin signatures reflect the organism’s level of response to the disease. Our data regarding changes in oxylipins at the system level show that oxylipin profiles can be used to evaluate the early stages of breast cancer.

## 1. Introduction

Viewing cancer as a systemic disease involving an interplay between the transformed cancer cells and the surrounding microenvironment and also as an all organism response for disturbance of the innate immunity system is currently a focus of investigation. Inflammation and genome instability underlie the hallmarks of cancer [1]. Oxylipins are signaling mediators that are deeply involved in innate immunity responses, the regulation of inflammatory responses including acute and chronic inflammation, tissue repair and other disturbances related to system diseases [2,3,4,5]. When produced as the response for stimuli, these oxidized lipids are responsible for inflammation and its subsequent resolution with an eventual return to pre-inflammation levels [4,5]. Unresolved chronic inflammation, characterized by abnormal oxylipins synthesis, becomes fertile soil for malignant transformation and tumor immune evasion [6,7,8].

In cancer, the role of oxylipins has been examined for many years, and a tumor promoting role of these substances was previously generally accepted (rev. in [6]). This was due to the fact that mainly derivatives of arachidonic acid (AA) along the COX pathway (mainly prostaglandin E2, PGE2) were studied [6]. In addition, studies have focused on eicosanoids produced by cancer cells or their microenvironment (rev. in [6]). A look at oxylipins as part of innate immunity responsible for body homeostasis after disturbances has presented new challenges. When considering this group of substances, it is important to take into account their heterogeneity and multiple roles in inflammatory processes. Indeed, oxylipins are synthesized from omega-3 or omega-6 polyunsaturated fatty acids (PUFAs) via three major pathways, named according to their respective key pathway enzymes: cyclooxygenase (COX), lipoxygenase (LOX), cytochrome P450 monooxygenase (CYP450) branches and also via anandamide (AEA) pathways and non-enzymatic conversions of PUFAs [2,3,4,5,6].

Eicosapentaenoic (EPA) and docosahexaenoic acid (DHA) omega-3 PUFAs, as well as their derivative oxylipins, hydroxyeicosapentaenoic acids (HEPEs), hydroxydocosahexaenoic acids (HDoHEs) and resolvin D1 (RvD1), are regarded as anti-inflammatory mediators [2,4]. Arachidonic acid (AA), an omega-6 PUFA, is mainly the source of prostaglandins (PGs), thromboxane (TX), leukotrienes (LTs) and hydroxyeicosatetraenoic acids (HETEs), referred to as the groups of pro-inflammatory oxylipins. Cyclopentenone PGs, non-enzymatic metabolites of PGE2 and PGD2, exhibit anti-inflammatory properties [4,5]. Oxidative derivatives of dihomo-γ-linolenic acid (DGLA) can be transformed into hydroxyeicosatrienoic (HETrE) acids or others [2]. Linoleic acid (LA)-derived oxylipins, such as hydroxyoctadecadienoic acids (HODEs), agonists of PPARγ, dihydroxyoctadecamonoenoic acids (DiHOMEs) or epoxyoctadecamonoenoic acids (EpOMEs), which are cytotoxic, exhibit both pro- and anti-inflammatory features [2,4,5].

So, oxylipins are synthesized simultaneously from different PUFAs and have various functions at the cellular level. There is still no clear explanation of how cells receive and respond to these multiple signals [3,9]. Oxylipin synthesis should not be studied in groups of separate substances, but in terms of oxylipin profiles, which can characterize the different states of the studied organisms.

Recently mass-spectrometry approaches to oxylipin profile detection gave the possibility to study the oxylipin biology both in the context of investigations of pathological processes and considering them as a potential biomarkers for a wide range of diseases, such as glaucoma [10], neurological pathologies [5,11], diabetes [12], oncology [6,8,13,14], and other inflammatory-based disturbances [12]

BC is the leading cause of cancer death among women worldwide. It originates from cells of the glandular epithelium of the mammary gland and is an extremely heterogeneous disease. Heterogeneity is observed both within a tumor from a single patient and between patients. This factor determines the risk of disease progression and its resistance to therapy [15]. The pathogenesis of BC is still unknown, along with the challenge of early diagnosis, which is a key to successful therapy. Modern approaches in genomics [16], transcriptomics [17] and metabolomics [18] are proposed for solving these questions. Although metabolomics may be considered a helpful tool in diagnosing early BC [18], there is few data on the use of oxylipin profiles for these purposes.

We found three works where oxylipin profiles of patients with BC were detected [13,14,19]. Oxylipin profiles of plasma samples of limited group BC patients without data concerning stages of disease and other important information about patients [13] or oxylipin profiles in tumor tissues of BC patients [14,19] show only possibility for promising results, but not answer the question concerning opportunity of blood oxylipin profiling disturbance reflects BC pathology. Therefore, we compared the signatures of the plasma oxylipin profile in patients with breast cancer (*n* = 162) and healthy volunteers (*n* = 153). Currently, the literature has accumulated a large amount of transcriptomic data of patients with breast cancer, and evidence of pathologically significant single nucleotide polymorphisms (SNPs) associated with breast cancer, which makes it possible to compare the data obtained during the metabolomic analysis and the corresponding changes at the gene level. It is known that expression of oxylipin metabolism genes is associated with cancer, and SNPs in oxylipin metabolism genes play an important role in various types of cancer [20,21,22,23]. An approach to compare transcriptome and metabolome data for biochemical pathways has been proposed previously [24,25], but it has not been used to analyze the oxylipin profiling. Therefore, we compiled a list of genes that may be associated with the synthesis or mechanism of action of the detected oxylipins [3,26] and analyzed changes in their expression. Evidence for an interaction between genetic polymorphisms and oxylipin levels is strong [20,22,27] and well shown for colorectal cancer [21,23]. Numerous data were focused mainly on the study of individual genes associated with the transport, elongation and desaturation of polyunsaturated fatty acids [20,21,22,27]. Polymorphisms of different genes can contribute to blood oxylipin profiles, but a comprehensive assessment of the oxylipin metabolism gene polymorphism has not been carried out. Such analysis gives a possibility to predict the most promising genes for further study. Therefore, in order to assess which cells or tissues may reflect changes in blood oxylipins, we analyzed the transcriptomes obtained previously in tissues and blood cells of BC patients and SNP data from databases for genes related to oxylipin metabolism.

## 2. Materials and Methods

### 2.1. Reagents and Internal Standards

High-performance liquid chromatography (HPLC)-grade acetonitrile (cat. no. 701881), methanol (cat. no. 701091) and water (cat. no. 7732-18-5) were procured from PanReac ApplyChem. The oxylipins standards were as follows: 6-keto PGF1α-d4 (cat. no. 315210), TXB2-d4 (cat. no. 319030), PGF2α-d4 (cat. no. 316010), PGE2-d4 (cat. no. 314010), PGD2-d4 (cat. no. 312010), LT C4-d5 (cat. no. 10006198), LTB4-d4 (cat. no. 320110), 5(S)-HETE-d8 (cat. no. 334230), 12(S)-HETE-d8 (cat. no. 334570), 15(S)-HETE-d8 (cat. no. 334720), oleoyl ethanolamide-d4 (cat. no. 9000552), EPA-d5 (cat. no. 10005056), DHA-d5 (cat. no. 10005057), and AA-d8 (cat. no. 390010) (Cayman Chemical, Ann Arbor, MI, USA). An Oasis^®^ PRIME hydrophilic-lipophilic balance (HLB) solid-phase lipid extraction cartridge (60 mg, 3 cc, cat. no. 186008056) was obtained from Waters, Eschborn, Germany.

### 2.2. Ethics Statement

The study was reviewed and approved by the Ethics Committee of P.A. Hertsen Moscow Cancer Research Institute, Ministry of Health of the Russian Federation (protocol #534, 7 March 2020) according to the guidelines approved under this protocol (Article 20, Federal Law “Protection of Health Right of Citizens of Russian Federation N323-FZ, 21 November 2011). The patients/participants provided their written informed consent to participate in this study.

### 2.3. Study Population

A group of Caucasian women ranging from 24–82 years old were recruited between July 2020 and September 2021. Exclusion criteria included: (1) acute infectious state, (2) diabetes, (3) autoimmune diseases. The study involved 169 BC patients and 152 HC. Considering the reported serum oxylipin variety during daytime [28], all blood sample collection was conducted in the morning in the fasted state. The plasma was obtained immediately after blood sampling, aliquoted and stored at −80 °C for further analysis.

### 2.4. Sample Preparation and UPLC-MS/MS Conditions

Samples were prepared for MS analysis by the solid-phase extraction (SPE) method using an Oasis^®^ PRIME HLB cartridge (60 mg, 3 cc) and VacElut Cartridge Manifold (Agilent, Santa Clara, CA, USA). For the identification of lipid mediators, the respective lipid extracts were analyzed using an 8040 series UPLC-MS/MS mass spectrometer (Shimadzu, Tokyo, Japan) in multiple-reaction monitoring mode at a unit mass resolution for both the precursor and product ions, as described previously [11]. The studied metabolites were identified and quantified according to the comparison of their multiple reaction monitoring parameters, retention times and peak areas with the parameters obtained for deuterated internal standard compounds of the same classes, using a commercial software method package Lipid Mediator Version 2 (Shimadzu, Tokyo, Japan), according to the manufacturer’s instructions.

### 2.5. Gene Expression Analysis and SNP Analysis

Salmon software (v1.0.0) was used for fast transcript quantification from RNA-seq data, taking reference human transcriptome GRCh38. R tximport was utilized for summation of expression levels at gene. Differential gene and transcript analyses were performed using DESeq2 and stageR. Genes with padj < 0.05 and log2FoldChange modulus > 1 were considered as differentially expressed. Then, filtration of resulting rosters was conducted, relying on the list of target genes. To identify the gene signature of genes involved in oxylipin biosynthesis, we performed a systematic search for transcriptomes using the previously developed tool ARGEOS [29]. The following criteria were used for the selection of datasets: the presence of control samples and samples with BC, host-human, total/polyA RNA, more than 10 samples, free access, material obtained directly from patients. A total of 10 datasets were selected for analysis (Table S3). The datasets were divided into tissue and blood samples. Next, we examined the expression of 33 selected genes using transcriptomics data (Figure S7). BC-related genes and SNP were retrieved from the DisGeNET database [30].

### 2.6. Experimental Data Analysis and Statistics

All studied samples were normalized on internal standards. Significantly changed oxylipins and biochemical blood markers were identified by pairwise two-sided *t*-tests with the following Bonferroni–Holm correction for multiple comparisons: adjusted *p*-value < 0.1, FC > 0.5. We used the partial least square discriminant analysis (PLS-DA) algorithm to investigate class separation. In this PLS-DA model feature, importance was estimated based on influence on projection (variable influence on projection; VIP score). The variable was considered important if its VIP score on at least one component was greater or equal to 1.5 (Metabolomics Standard Initiative, level 1).

## 3. Results

### 3.1. Clinical Characteristics

The study involved 169 BC patients and 152 HC. The anthropometric and demographic parameters of the enrolled individuals are presented in Table 1. In total, 53 patients had the LumA form, 87-Lum B (including 5 HER2+), 4 HER2+, 10 TH and 15 unidentified subtypes.

### 3.2. Identification of Altered PUFAs and Oxylipins in Healthy Donor and Breast Cancer Patients

We characterized the PUFAs and oxylipin profiles of human plasma samples for HC and BC using UPLC-MS/MS. We detected a total of 34 metabolites in human plasma (Table S1). Metabolites were from different lipid classes: 4 PUFA (AA, DHA, dihomo-γ-linolenic acid (DGLA) and EPA), 14 AA derivatives, one DGLA derivate, 4 DHA derivatives, 1 α-linolenic acid (ALA) derivative, 2 EPA derivatives, 7 linoleic acid (LA) derivatives and non-PUFA-derived compound oleic acid (OEA).

**Figure 1:**
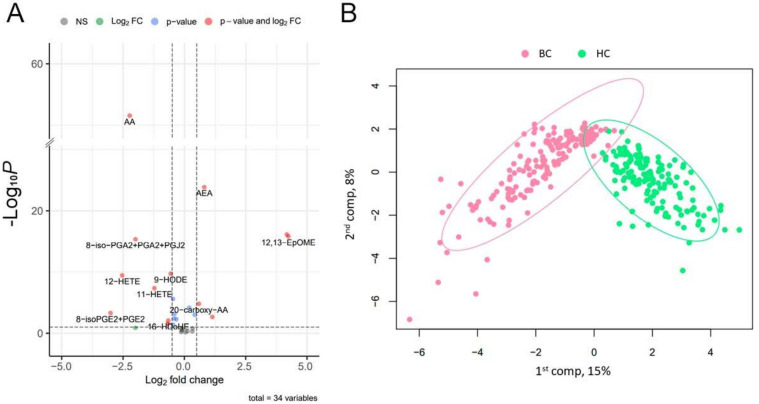
Concentration of oxylipin changes in BC patients. (**A**) Volcano plot indicating significantly changed compounds. X-axis indicates a log2 fold change of BC to HC patients. Y-axis indicates −log10 *p*-values (adjusted). The cut-off for *p*-values is indicated based on Bonferroni correction. Compounds that changed insignificantly are indicated in gray, compounds whose means changed in BC (relative to HC) more than twofold or less than twofold, but insignificantly, are indicated in green. Red dots stand for compounds that changed more than twofold and had a *p*-value (adjusted) < 0.05. (**B**) PLS-DA model discriminating against HC and BC. The explained variance of each component is indicated in brackets on the corresponding axis.

To evaluate the separate metabolites that differ among BC and HC groups, we performed pairwise comparisons of normalized metabolite concentrations. The results were then illustrated using a volcano plot with Holm–Bonferroni correction (Figure 1A). The 18 metabolites with significantly changed concentrations were detected (Table 2). Barplots of the indicated compounds’ relative concentrations are presented in Figure S1.

**Figure 2:**
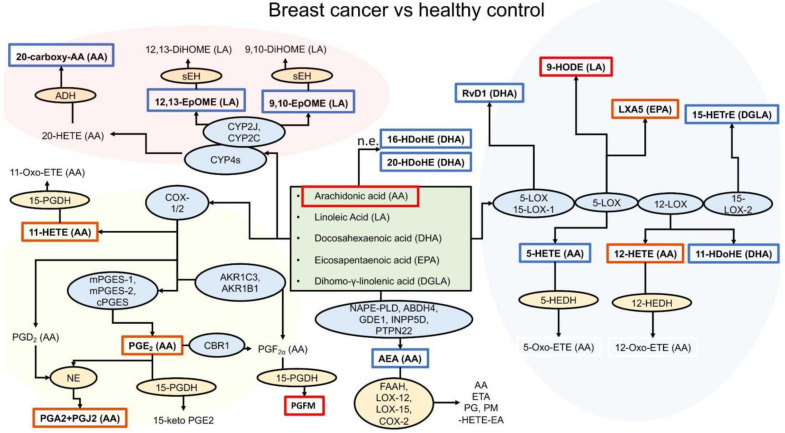
Schema of oxylipin profiles for breast cancer and healthy control donors. Cyclooxygenase (COX), lipoxygenase (LOX), cytochrome P450 monooxygenase (CYP450) branches and also anandamide (AEA) pathways and non-enzymatic conversions of PUFAs are noted. Analyzed oxylipins are marked with a red frame for induced in BC and a blue frame for reduced in BC samples. Abbreviations: Metabolites: AA—Arachidonic acid, HETE—hydroxyeicosatetraenoic acids, HETrE—hydroxyeicosatrienoic acids, AEA—anandamide, HdoHE—hydroxydocosahexaenoic acids, EpOME—epoxyoctadecamonoenoic acids, PGA2—prostaglandin A2, PGJ2—prostaglandin J2, HODE—hydroxyoctadecadienoic acids, LXA5—lipoxin A5, 20-carboxy-AA—20-carboxy arachidonic acid, 13,14-dihydro-15-keto-PGF2a—13,14-dihydro-15-keto prostaglandin F2α, PGE2—prostaglandin E2. Proteins: LOX—lipoxygenase, 5-HEDH—5-hydroxyeicosanoid dehydrogenase, NAPE-PLD—NAPE-specific phospholipase D, ABDH4—α/β-hydrolase domain 4, GDE1—glycerophosphodiester phosphodiesterase 1, PTPN22—Protein Tyrosine Phosphatase Non-Receptor Type 22, INPP5D—inositol polyphosphate-5-phosphatase D, FAAH—fatty acid amide hydrolase, COX-2 —cyclooxygenase-2, PTGS2—prostaglandin-endoperoxide synthase 2, ALOX—arachidonate -lipoxygenase, CYP2J2—cytochrome P450 2J2, CYP2C—cytochrome P450 2C subfamily, mEH—microsomal epoxide hydrolase, –I—soluble epoxide hydrolase, EP–X—epoxide hydrolase, E–3—epoxide hydrolase 3, E–3—epoxide hydrolase 4, mPGES–1—microsomal prostaglandin E synthase-1, PG–S—prostaglandin-D synthase, CYP4A–1—cytochrome P450 4A11, CYP4–2—cytochrome P450 4F2, CYP4F–B—cytochrome P450 F3B, A–H—alcohol dehydrogenase, 15-PG–H—15-hydroxyprostaglandin dehydrogenase, AKR1–3—aldo-keto reductase family 1 member C3, AKR1–1—aldo-keto reductase family 1, member B1, CBR1- carbonyl reductase 1, n–e—non-enzymatically.

For testing whether BC and HC could be distinguished based on oxylipin concentrations, PLS-DA was performed. The model was evaluated via cross-validation based on the overall error, BER and area under curve (AUC) values (Figure S2). The optimal number of components was three. Projections on the first two components are presented in Figure 1B. Studied groups were separated with a small overlap. For each metabolite, the VIP score was estimated. The value of this parameter addresses the explained variation between classes in each projection. A total of five metabolites (11-HdoHE, 5-HETE, 15-HETrE, AEA and AA) with VIP score values > 1.5 are shown in Table 3. The resulting oxylipin analysis scheme is shown in Figure 2.

Data show that the synthesis of AA and its COX-derived metabolites, namely, PGA2 + PGJ2, 11-HETE and PGE2, is increased in BC patients (Table 2, Figure 2). There is no increase in PGF2a, but we detected an increase in its main metabolite 15-keto-13,14-dihydro-PGF2a (PGFM), which is formed from PGF2a by 15-PGDH (15-hydroxyprostaglandin dehydrogenase; HPGD) [2,3,4,5]. The latter is used to measure in vivo PGF2a biosynthesis.

Of the metabolites synthesized by the LOX pathway, we observed an increase in 12-HETE (AA-derivative), 9-HODE (LA-derivative) and LXA5 (lipoxin A5, EPA-derivative). We observed a decrease in DGLA-derivative 15-HETrE, DHA-derivatives resolvin D1 and 11-HDoHE, formed enzymatically by the LOX pathway. Interestingly, the LOX pathway derivatives of DHA decreased, while the 16-HDoHE and 20-HDoHE derivatives formed non-enzymatically from DHA increased.

Of the metabolites synthesized by the cytochrome P450 (CYP) pathway, we observed a decrease in 9,10-EpOME and 12,13-EpOME (LA metabolites). We did not detect 20-HETE in plasma, but we found a consistent decrease of 20-carboxy arachidonic acid (20-COOH-AA), which is considered a product of 20-HETE-derivative oxidized by alcohol dehydrogenase [31]. At the same time, we observed a decrease in AEA.

### 3.3. Stage, Molecular Subtype, Higher Body Mass Index (BMI) and Blood Biochemical Marker Correlation with Oxylipin Concentrations

At the next stage, we compared whether there is a correlation between the stages of the patient’s disease, molecular subtype of BC, higher body mass index (BMI) and blood biochemical marker correlation with oxylipin concentrations. No correlations were observed between compared parameters (detailed in Figures S3–S6).

### 3.4. Pathway Integration of Oxylipin Mediator with Gene Expression

We have compiled a list of genes involved in the metabolism of targeted oxylipins based on known data [2,3,4,26]. A total of 33 genes were selected, presented in Table S2.

#### 3.4.1. DEGs in Tissue Datasets BC vs. HC

In the GSE141142 dataset, the expression of the ALOX12B genes was increased, in the GSE52194 dataset: the expression of a number of cytochromes—CYP2C19, CYP2C9 CYP4F2, CYP4F3, and the HPGDS, NAPEPLD, PTPN22 genes was increased. Reduced activity of three epoxide hydrolases EPHX1, EPHX2, EPHX3, as well as genes of prostaglandin biosynthesis: PTGES, PTGES3, PTGS2 was observed. In the dataset GSE100925 (tissue HC vs. tissue BC) and GSE80333 (fibroblasts from HC vs. fibroblasts from BC), none of the selected genes was DEG. In the GSE89225_conv dataset, the expression of ALOX15B is reduced.

#### 3.4.2. DEGs in Blood Datasets BC vs. HC

In the dataset, GSE111842 (peripheral blood from HC vs. circulating tumor cells sorted from peripheral blood from BC patients) and GSE148991 (plasma and buffy coat from HC vs. circulating tumor cells from metastatic BC patients), none of the genes were DEG. The GSE117970 dataset (monocytes from HC vs. monocytes from BC patients) showed an increase in AKR1C3, CYP2C9, EPHX2 and EPHX4, and a decrease in PTGES. In the GSE68086 dataset (the largest dataset in terms of the sample size—blood platelets from HC vs. blood platelets from BC patients), there was a decrease in the expression of genes AKR1B1, ALOX12B, ALOX15B, cytochromes CYP2C19, CYP4A11, CYP4F2, epoxide hydrolase EPHX4 and FAAH. The genes of the prostaglandin E2 biosynthesis—PTGES and PTGES2—were also reduced.

**Figure 3:**
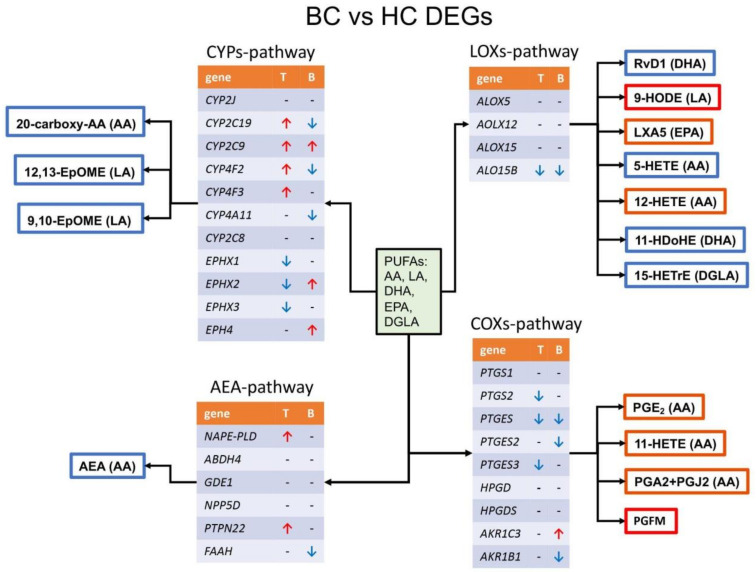
Schema of DEGs in tissue (T) and blood (B) BC patient transcriptomics datasets. Identified DEGs are marked with a red arrow for induced genes in BC and a blue arrow for reduced genes in BC patients. Abbreviations: Genes: LOX—lipoxygenase, 5-HEDH–5-hydroxyeicosanoid dehydrogenase, NAPE-PLD—NAPE-specific phospholipase D, ABDH4—α/β-hydrolase domain 4, GDE1—glycerophosphodiester phosphodiesterase 1, PTPN22—protein tyrosine phosphatase non—receptor type 22, INPP5D—inositol polyphosphate-5-phosphatase D, FAAH—fatty acid amide hydrolase, COX-2—cyclooxygenase-2, PTGS2—prostaglandin-endoperoxide synthase 2, ALOX—arachidonate -lipoxygenase, CYP2J2—cytochrome P450 2J2, CYP2C—cytochrome P450 2C subfamily, mEH—microsomal epoxide hydrolase, she—soluble epoxide hydrolase, EPHX—epoxide hydrolase, EH3—epoxide hydrolase 3, EH3—epoxide hydrolase 4, mPGES-1—microsomal prostaglandin E synthase-1, PGDS—prostaglandin-D synthase, CYP4A11—cytochrome P450 4A11, CYP4F2—cytochrome P450 4F2, CYP4F3B—cytochrome P450 F3B, ADH—alcohol dehydrogenase, 15-PGDH—15-hydroxyprostaglandin dehydrogenase, AKR1C3—aldo-keto reductase family 1 member C3, AKR1B1—aldo-keto reductase family 1, member B1, CBR1- carbonyl reductase 1, n.e—non-enzymatically.

Taken together, DEG analysis of tissue and blood samples from BC patients revealed that 19 genes for oxylipin biosynthesis change their expression level (Figure 3). For tissue samples, a decrease in the expression of EPHs, genes of prostaglandin biosynthesis and an increase in the expression of CYPs were observed. For blood samples, a decrease and an increase in various CYPs and EPHs and a decrease in the expression of a number of the prostaglandin biosynthesis genes were observed. So, we did not observe a direct correlation between the oxylipin profile and changes at the gene expression level.

### 3.5. Gene-Disease Associations

**Figure 4:**
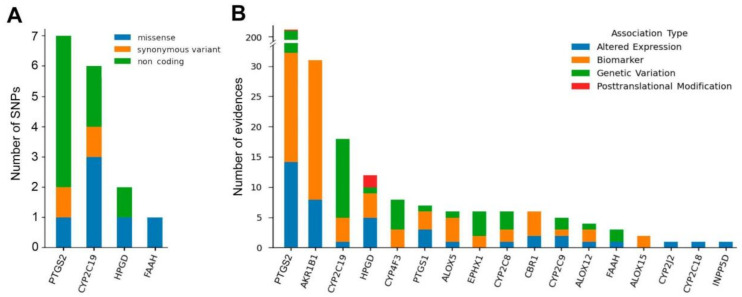
SNPs (**A**) and evidence (**B**) associated with BC for genes involved in oxylipin biosynthesis.

SNPs significantly affect the metabolism of PUFAs [20,27]. and SNPs in oxylipin metabolism genes play an important role in various types of cancer [21,22,23]. Therefore, we analyzed the presence of SNPs in genes involved in the biosynthesis of oxylipins that change when comparing BC with HC. To do this, we analyzed the list of selected genes (Table S4) using the DisGeNet service (https://www.disgenet.org/ (accessed on 1 February 2022)) [30]. The service allows users to obtain information about the associations between variation in a gene and the risk of developing a disease based on the analysis of article texts, as well as verified databases, including UniProt, ClinVar, the GWAS Catalog, and GWAS db. The resulting list of SNPs in the target genes was verified manually, and non-significant values were excluded from the analysis. It was found that, from the list of genes encoding the biosynthesis of oxylipins, there are SNPs in four genes: PTGS2 (COX-2 enzyme), CYP2C19 (cytochrome P450 2C19), HPGD (15-hydroxyprostaglandin dehydrogenase) and FAAH (Figure 4A).

In the coding regions, synonymous and nonsynonymous SNPs are distinguished. Synonymous SNPs do not result in amino acid substitutions and usually do not affect enzyme activity. In the context of association with diseases, nonsynonymous SNPs play an important role, in which a single amino acid residue substitution (missense) occurs, or a stop codon (nonsense) is formed prematurely. In non-coding regions, SNPs are observed in introns and intergenic regions, particularly in the regulatory areas and binding sites of protein factors. Functional SNPs can enter splice sites and regions that control transcription and thus affect gene expression. A separate category comprises non-coding SNPs that affect the structure and functioning of different types of RNA.

DisGeNet programs also enable the detection of disease-related conditions associated with baseline data analysis from different databases. We analyzed an association between selected genes (Table S4) and BC. The service highlights: altered expression (alterations in the function of the protein by means of altered expression of the gene are associated with the disease phenotype); biomarker (the gene/protein either plays a role in the etiology of the disease (e.g., participates in the molecular mechanism that leads to disease) or is a biomarker for a disease); genetic variation (used when a sequence variation (a mutation, an SNP) is associated to the disease phenotype, but there is still no evidence to say that the various causes the disease. In some cases, the presence of the variants increases the susceptibility to the disease, post-translational modification (alterations in the function of the protein by means of post-translational modifications (methylation or phosphorylation of the protein) are associated with the disease phenotype. It was found that for 17 genes, such an association is present (Figure 4B), the color indicates the type of association for the observed genes. The y-axis represents the number of gene references in a BC context. The most common genes are cytochromes, such as CYP2C19, CYP4F3, CYO2C8 and CYP2C9, and oxylipin degradation genes such as HPGD, EPHX1 and CBR1.

In terms of the volume of evidence, the largest number of associations is observed for the PTGS2 gene (210) and AKR1B1 (31), while they are mainly associated with BC biomarkers. CYP2C19, PTGS2, HPGD and FAAH are included in both the list of genes associated with BC and the list of SNPs associated with BC.

## 4. Discussion

In the present study, we used a multi-platform approach combining UPLC-MS/MS metabolomics data, DEG gene expression datasets from open databases and BC-associated SNP information to examine characteristic changes in plasma oxylipin profile in patients with BC.

Physiological activities of oxylipin mixtures have begun to be studied relatively recently [9,11,13,32]. Platelets [33], T-cells [34], monocytes [35], and vascular endothelium [36] can contribute to the oxylipin profile in blood plasma. There is no doubt that lipids, including oxylipins, are altered in various types of tumors (rev. [37]). It remains an open question whether the composition of oxylipins in the blood changes. If so, what might the oxylipin profiles show?

We compared our data with others where we measured oxylipin profiles in the blood of patients with various types of cancer (Table S4). The authors use different sets of oxylipins for detection, so it is not easy to compare. We observed 18 compounds and 13 of them were presented in other panels of detection. These are the compounds that were looked at in at least one of the presented works. The data show that the profile changes with the type of cancer. In a large dataset (*n* = 157) in the blood of patients with ovarian cancer, an increase in the oxidized derivatives of LA-9-HODE, 13-HODE and 12,13-DiHOME—was observed, while no changes were detected in AA derivatives, such as PGE2 or -HETE [38]. An increase in AA derivatives such as 11-HETE, 5-HETE, 15-HETE was observed in the blood of patients with lung cancer (*n* = 37) [39] and prostate cancer (*n* = 19) [40]. Interestingly, the concentration of free AA decreased in the case of prostate cancer [40] and increased in the case of lung cancer [39], as well as our data for BC. When analyzing the blood oxylipins of patients with colorectal cancer (*n* = 25), a decrease in 12-keto-LTB4 and LA-derivatives 9-HODE and 13-HODE was observed [41]. The comparison of oxylipin profiles for different types of cancer supposed that changes in the oxylipin metabolism are specific characteristics for each type of cancer studied.

We observed an increase in the concentration of arachidonic acid (AA) derivatives synthesized via the cyclooxygenase (COX) pathway, a decrease in the concentration of metabolites synthesized via the epoxygenase pathway (AA and linoleic acid (LA) metabolites), lipoxygenase (LOX) pathway (DHA metabolites), and a decrease in the concentration of endogenous cannabinoid Anandamide (AEA) in the blood of BC patients. This is consistent with the data obtained for the individual compounds such as PGE2 or LOX metabolites of linoleic acid and linolenic acids [13,42]. The advantages of our work are that we simultaneously looked at a large number of oxylipins (profiles) and did so in a large sample of data. In addition, we observed changes in compounds that were not previously analyzed in the profiles, although they are considered important. Among them is AEA, which is a cannabinoid that demonstrates anti-cancer properties in various cancer models, including breast cancers [43]. Several lines of evidence support that tumor development and progression may be associated with disturbances of the endocannabinoid balance [44]. Our data are in accordance with others, it can be assumed that the spectrum of action of oxylipins is shifted towards a pro-inflammatory response, since there is an increase in PGE2, a decrease in AEA and other derivatives with anti-inflammatory properties. Our data support the assumption that oxylipin changes reflect the general status of the organism and may not directly reflect changes observed in a specific tumor tissue. In any case, the oxylipin blood profile itself has characteristic features for each disease (Table S4).

The transcriptome part of our study sheds new light on the role of oxylipins in tumor diseases. We found that many genes of oxylipin metabolism change their expression when comparing BC with HC. Studying the data obtained from tumor tissue, we noted increased expression of cytochromes and decreased epoxide hydrolases, which indicates the possibility of increasing the concentration of 12,13-EpOME and 9,10-EpOME (Figure 3). Similar data were obtained when measuring oxylipins in the tissues of BC patients [14]. Our analysis showed a decrease in the expression of genes for the biosynthesis of prostaglandins (COX-2, prostaglandin synthases) and an increase in the expression of HPGDS (prostaglandin D synthase) (Figure 3). These changes can lead to a decrease in the concentration of PGE2 and an increase in other products such as 11-HETE, PGFM or PGD2 (Figure 3). Increases in 11-HETE and PGD2 were observed in BC tissue [14]. An increase in N-acyl phosphatidylethanolamine phospholipase D (NAPEPLD), a main enzyme of AEA synthesis [43], should lead to an increase in AEA. This is consistent with the data that high levels of the AEA precursor, N-acylphosphatidylethanolamine in BC tissue were observed [45]. Analysis of DEGs shows that tumor tissue samples or blood cells from BC patients have their own expression pattern of oxylipin metabolism genes. These changes do not directly correlate with changes in the oxylipin profile in blood plasma. Blood oxylipin profiles are an independent characteristic for distinguishing the early stages of BC in comparison with HC.

At present, there is no doubt about the importance at different stages of cancer of changes in individual AA-derivatives of oxylipins [6,46,47] or in CYPs, COXs and LOXs, enzymes for the oxylipins synthesis [14,48]. Our work shows changes in the oxylipin profile in BC compared to HC blood. This characteristic remains unique and does not directly reflect changes in the transcription of genes for the metabolism of oxylipins in blood cells or tumor tissues. Many questions remain: Do oxylipin changes reflect cancer stages or specific body defense responses? Is the observed change protection against disruption of homeostasis in a disease or a manifestation of pathology? The answer to this question influences the direction of therapeutic strategies. Other questions include: Could oxylipin profiles be used to diagnose specific cancer and its stages? Could blood oxylipins specifically reflect changes in tissue pathology? Although most of the questions on oxylipin biology remain unsolved, our work answers in the affirmative these last two questions. The question remains as to what changes exactly reflect the oxylipin blood profile. Although it has been suggested that changes in metabolites reflect changes in tumor tissues [17], our data indicate that it is more likely that changes in blood oxylipins reflect the body’s systemic response to pathology, not directly related to tumor or changes in blood cells. Further studies will show how sustainable this characteristic is, but at present, the data show that the oxylipin blood profile can be used to detect early stages of BC compared with HC.

## 5. Conclusions

To understand the biology of cancer, it is very important to elucidate the mechanisms of involvement in this process of oxylipins, which are key participants in the innate immune system. Our study shows systemic changes in PUFAs metabolism in breast cancer, and reveals the potential of oxylipin profiling to evaluate the early stages of breast cancer.

## Figures and Tables

**Table 1:** Demographic parameters of patients and disease characteristics.

Breast Cancer Patients (*n* = 169)		
	Mean	SD
Age [yrs]	54.5	12.6
Body mass index (BMI)	26.8	5.8
		*n*
Clinical cancer stage	0	12
	I	118
	IIA	31
	IIB	8
Molecular subtype	LumA	53
	LumB	82
	LumB, Her2+	5
	Her2+	4
	TN	10
	-	15
**Healthy Controls (*n* = 152)**		
	**Mean**	**SD**
Age [yrs]	49.8	10.1
Body mass index (BMI)	27.3	5.2

Abbreviations: LumA—Luminal A, LumB—Luminal B, HER2+—human epidermal growth factor receptor 2 (HER2) positive breast cancer, TN—triple-negative breast cancer.

**Table 2:** Summary of the 18 identified metabolites and their statistical values.

Metabolite	log2FC	Source	*p*-Value
AA	−2.2	AA	2.893548 × 10^−52^
5-HETE	5.1	AA	7.575180 × 10^−39^
15-HETrE	6.0	DGLA	1.448791×10^−35^
AEA	0.8	AA	1.387907 × 10^−24^
11-HDoHE	5.2	DHA	1.940721 × 10^−18^
9,10-EpOME	4.2	LA	7.414864 × 10^−17^
12,13-EpOME	4.2	LA	1.554750 × 10^−16^
PGA2+PGJ2	−2.0	AA	4.461561 × 10^−16^
9-HODE	−0.6	LA	1.883501 × 10^−10^
12-HETE	−2.5	AA	3.520998 × 10^−10^
11-HETE	−1.2	AA	4.466121 × 10^−8^
LXA5 (15-HEPE)	−6.15	EPA	8.651793 × 10^−6^
20-carboxy-AA	0.6	AA	1.564957 × 10^−5^
13,14-dihydro-15-keto-PGF2a (PGFM)	−5.4	AA	2.201848 × 10^−4^
PGE2	−3.0	AA	4.969367 × 10^−4^
Resolvin-D1	1.1	DHA	2.209084 × 10^−3^
16-HDoHE	−0.7	DHA	8.448198 × 10^−3^
20-HDoHE	−0.7	DHA	2.689864 × 10^−2^

Fold-changes are presented as the median average of cancer divided by control. False discovery rate-adjusted *p*-values using the Benjamini–Hochberg procedure are reported at the last column. Abbreviations: AA-arachidonic acid, HETE—hydroxyeicosatetraenoic acids, HETrE—hydroxyeicosatrienoic acids, AEA—anandamide, HDoHE—hydroxydocosahexaenoic acids, EpOME–epoxyoctadecamonoenoic acids, PGA2—prostaglandin A2, PGJ2—prostaglandin J2, HODE—hydroxyoctadecadienoic acids, LXA5—Lipoxin A5, 20-carboxy-AA—20-carboxy arachidonic acid, 13,14-dihydro-15-keto-PGF2a-13,14-dihydro-15-keto Prostaglandin F2α, PGE2–prostaglandin E2.

**Table 3:** VIP scores for five metabolites. A cutoff value of 1.5 is established for VIP selection.

Name	11-HDoHE *	5-HETE *	15-HETrE *	AEA *	AA *
VIP scores	1.518144	2.068291	2.007403	1.621081	2.309511

* *Volcano plot indicating significantly changed compounds, p < 0.05 (adjusted for multiple testing).* Abbreviations: HDoHE—hydroxydocosahexaenoic acids, HETE—hydroxyeicosatetraenoic acids, HETrE—hydroxyeicosatrienoic acids, AEA—anandamide, AA—arachidonic acid.

## Data Availability

Data available on request due to restrictions privacy or ethical.

## Supplementary Materials

The following supporting information can be downloaded at: https://www.mdpi.com/article/10.3390/cancers14082041/s1, Figure S1: Relative concentrations of separate oxylipins measured in BC patients in comparison with HCs; Figure S2: ROC (receiver operating characteristic) curve for chosen number of components (3) and the Balanced Error Rate (BER) and overall error rate estimated via cross-validation for different component numbers; Figure S3: Correlation between the stages of the patient’s disease; Figure S4: Stage and molecular subtype correlation with oxylipin concentrations; Figure S5: Higher body mass index (BMI) correlation with oxylipin concentrations; Figure S6: Blood biochemical markers correlation with oxylipin concentrations; Figure S7: Volcano plots indicating significantly changed genes; Table S1: UPLC-MS/MS parameters of the identified lipids; Table S2: Oxylipins changed in BC compared with HC patients, human enzymes and genes involved in lipid metabolism; Table S3: Datasets involved in the analysis of differential gene expression; Table S4. Changes in PUFA and human oxylipins in different types of cancer.

## Abbreviations

AA	Arachidonic acid
AEA	Anandamide
COX	Cyclooxygenase
CYP450	Cytochrome P450 monooxygenase
DHA	Docosahexaenoic acid
DiHOME	Dihydroxyoctadecamonoenoic acid
HC	Healthy control
HDoHE	Hydroxydocosahexaenoic acid
HETE	Hydroxyeicosatetraenoic acid
HODE	Hydroxyoctadecadienoic acid
LA	Linoleic acid
LOX	Lipoxygenase
PG	Prostaglandin
PUFAs	Polyunsaturated fatty acids
BC	Breast cancer
UPLC-MS/MS	Ultra-performance liquid chromatography-tandem mass spectrometry
LumA	Luminal A
LumB	Luminal B
HER2+	human epidermal growth factor receptor 2 (HER2) positive breast cancer,
TN	triple-negative breast cancer
