# Supplementary material for: Multi-Omics Approach Points to the Importance of Oxylipins Metabolism in Early-Stage Breast Cancer
Source: Cancers (Basel). 2022 Apr 18;14(8):2041. doi: 10.3390/cancers14082041 (PMC9032865; doi:10.3390/cancers14082041)
Supplement: Supplementary file 1 [file cancers-14-02041-s001.zip › cancers-1638014-supplementary.pdf]

cancers

Multi-Omics Approach Points to the Importance of Oxylipins Metabolism in Early-Stage Breast Cancer

Dmitry V. Chistyakov, Mariia V. Guryleva, Elena S. Stepanova, Lyubov M. Makarenkova, Elena V. Ptitsyna, Sergei V. Goriainov, Arina I. Nikolskaya, Alina A. Astakhova, Anna S. Klimenko, Olga A. Bezborodova, Elena A. Rasskazova, Olga G. Potanina, Rimma A. Abramovich, Elena R. Nemtsova and Marina G. Sergeeva

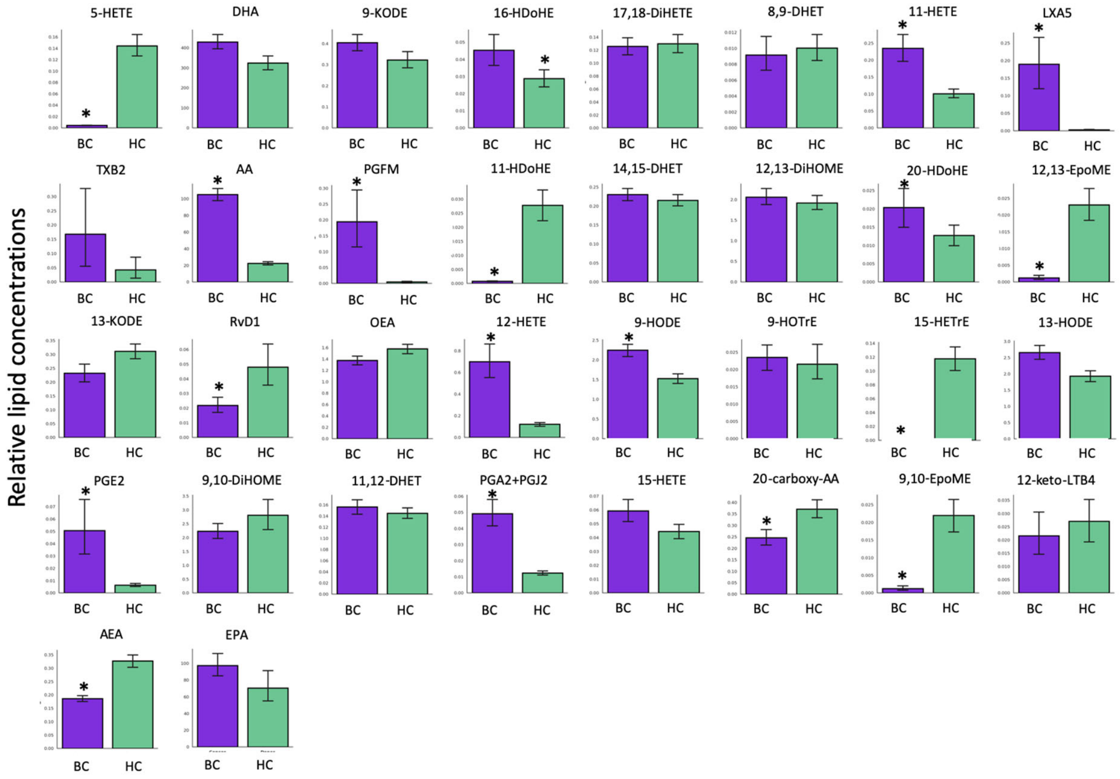

Figure S1. Relative concentrations of separate oxylipins measured in BC patients in comparison with HCs. Pairwise comparison of adjusted means was conducted taking into account the age and sex of patients. * $p < 0.1$ (adjusted for multiple testing).

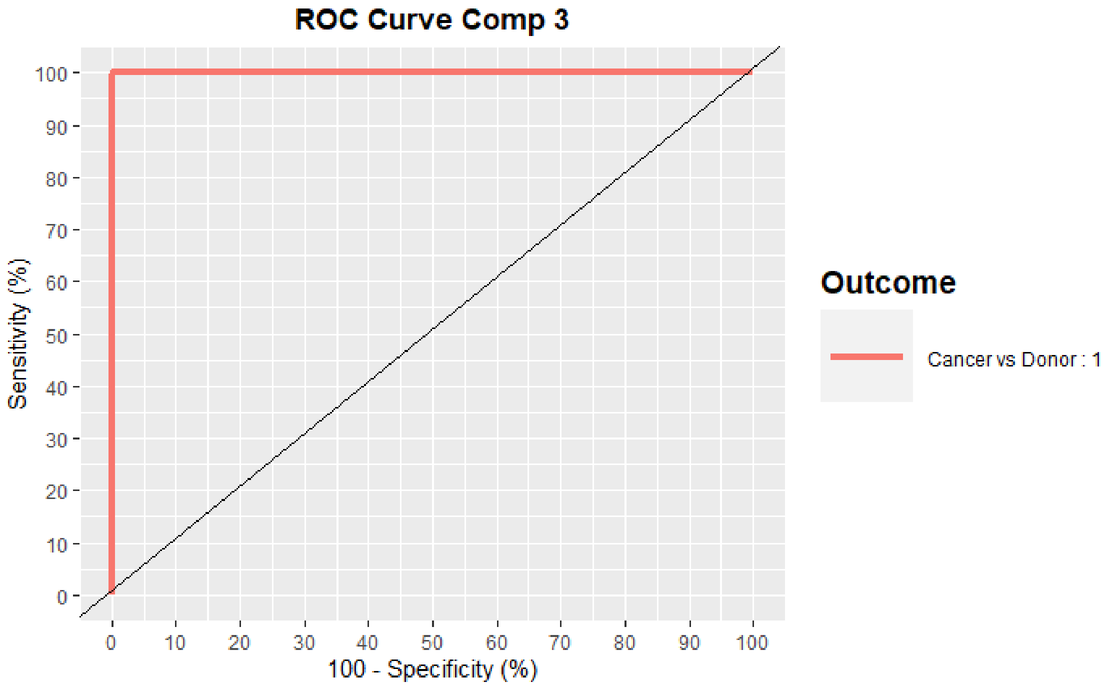

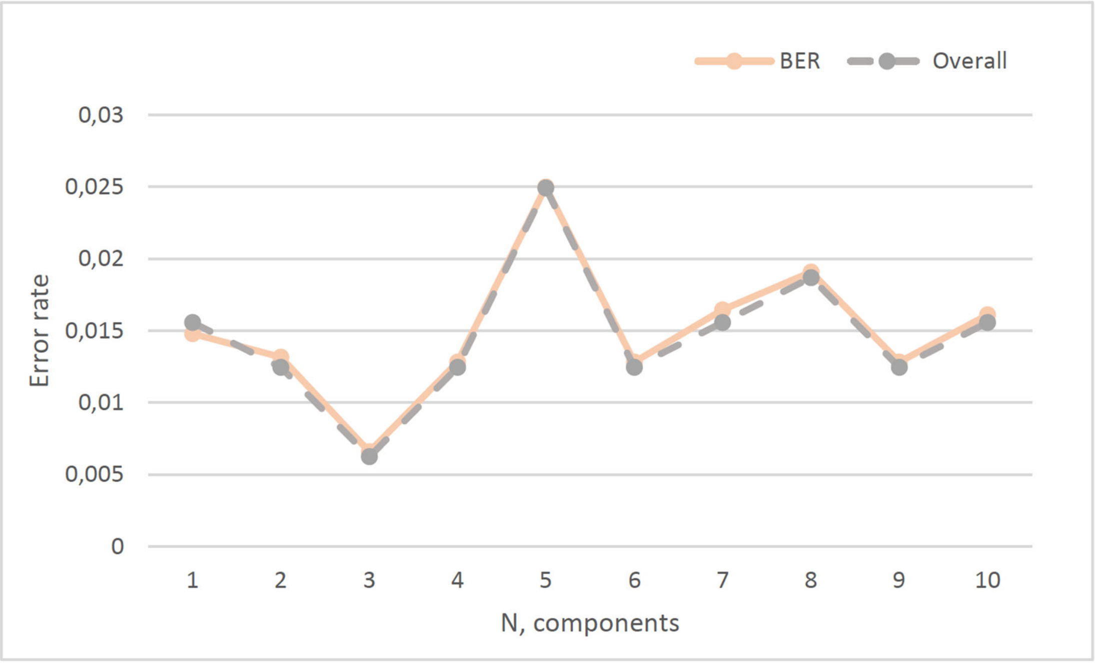

Figure S2. ROC (receiver operating characteristic) curve for chosen number of components (3) and the Balanced Error Rate (BER) and overall error rate estimated via cross-validation for different component numbers.

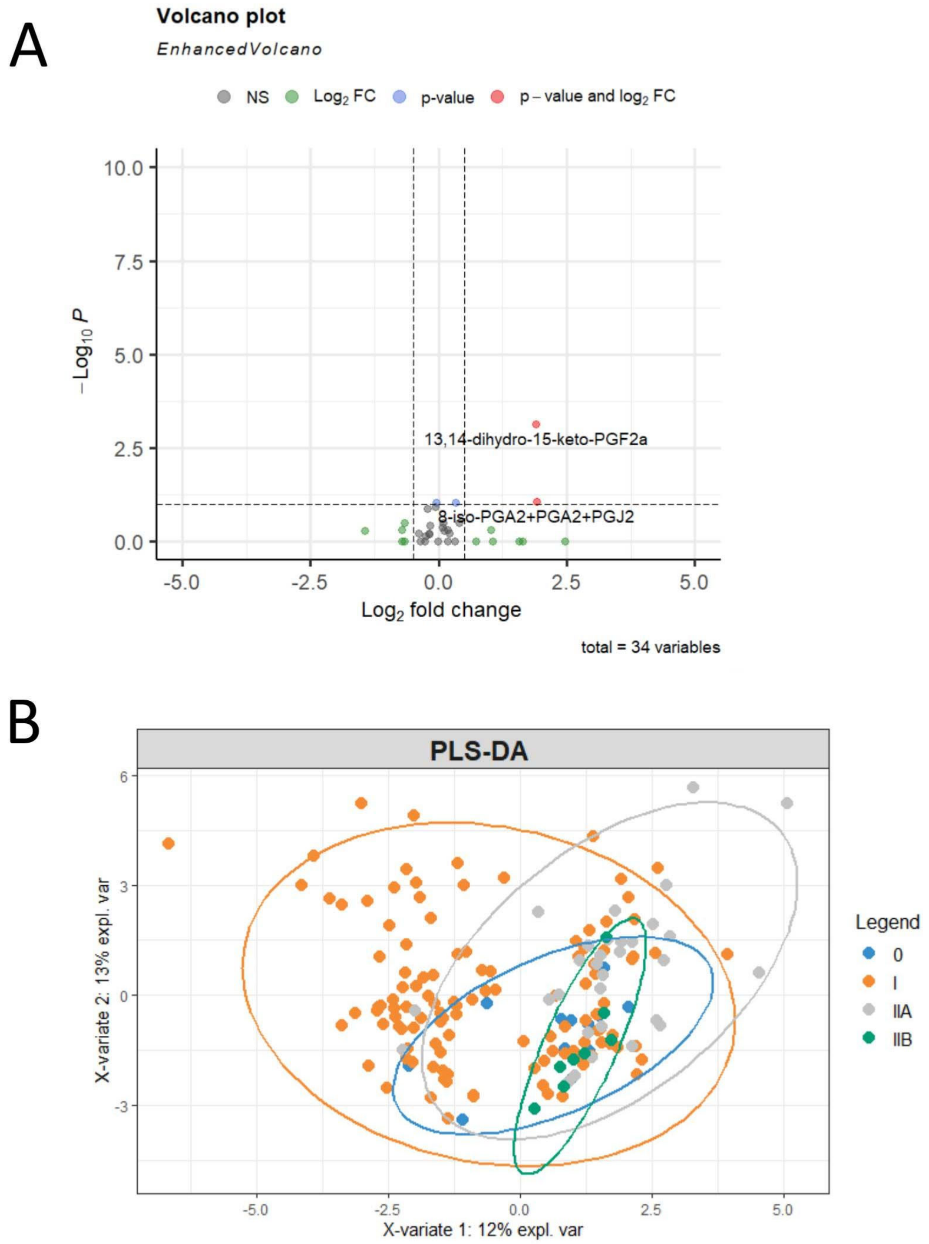

Figure S3. Correlation between the stages of the patient's disease (0 (n = 12), I/IA (n = 118), IIA (n = 31), IIB (n = 8)) and the concentration of oxylipins. Due to the imbalance in the sample groups, we merged groups IIA and IIB. The results were then illustrated using a volcano plot with Benjamini-Hochberg correction and PLS-DA. (**A**) Volcano plot indicating significantly changed compounds. The X-axis indicates a log2 fold change of Stage I vs (Stage IIA + Stage IIB) breast cancer patients. Y-axis indicates −log10 p-values (adjusted). The cut-off for p-values is indicated based on Benjamini-Hochberg correction. Compounds that changed insignificantly are indicated in gray, compounds whose means changed in BC (relative to HCs) more than twofold or less than twofold but insignificantly are indicated in green. Red dots stand for compounds, which changed more than twofold and had a p-value (adjusted) < 0.1. (**B**) The partial least square discriminant analysis (PLS-DA) model discriminating against 4 groups of BC patients. The explained variance of each component is indicated in brackets on the corresponding axis

A

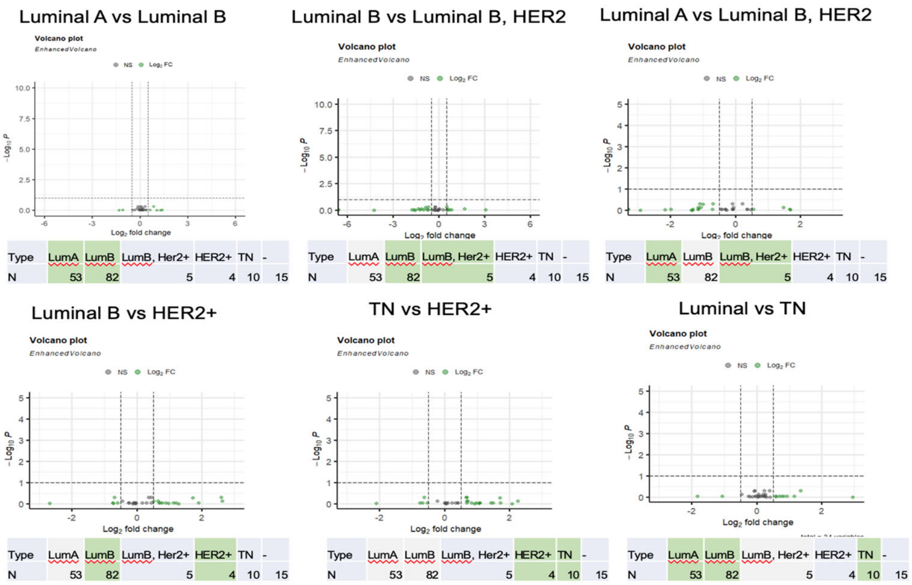

B

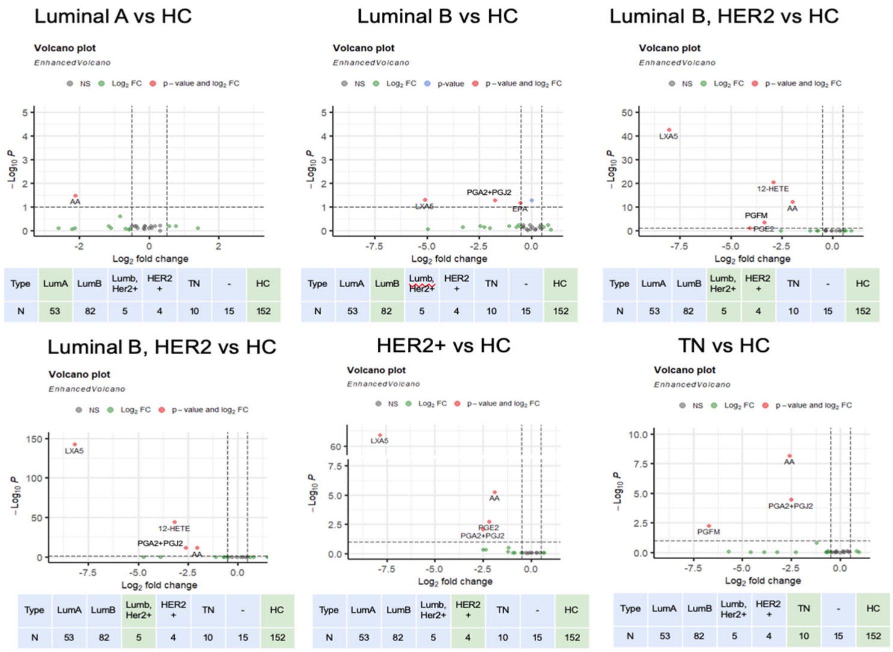

Figure S4. Stage and molecular subtype correlation with oxylipin concentrations. The following subtypes were presented in the analysis: luminal A (n = 53), luminal B (n = 82), luminal B, Her2+ (n = 5), Her2+ (n = 4), triple negative (n = 10). The X-axis indicates a log2 fold change of the compared groups, Y-axis indicates −log10 p-values (adjusted). The cut-off for p-values is indicated based on Benjamini-Hochberg correction. **(A)** Volcano plots indicate significantly changed compounds between BC subtypes. Compounds that changed insignificantly are indicated in gray, compounds whose means changed in the first BC subtype (relative to the other BC subtype) more than twofold or less than twofold significantly are indicated in green. Red dots stand for compounds, which changed more than twofold and had a p-value (adjusted) < 0.1. **(B)** Volcano plots indicate significantly changed compounds between BC subtype and HC. Compounds that changed insignificantly are indicated in gray, compounds whose means changed in the BC subtype (relative to HC) more than twofold or less than twofold significantly are indicated in green. Red dots stand for compounds, which changed more than twofold and had a p-value (adjusted) < 0.1

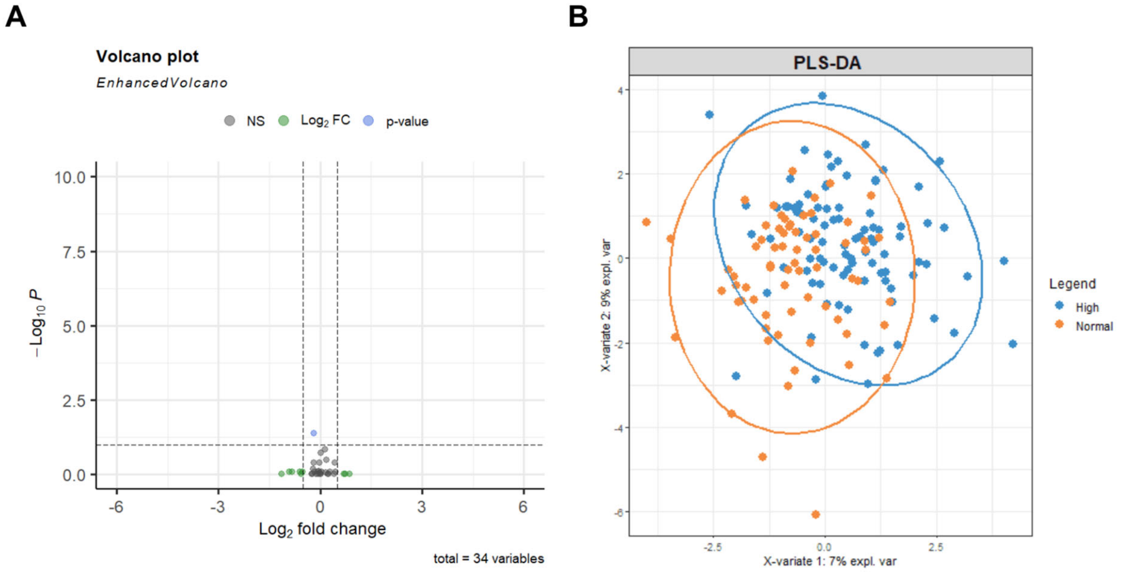

Figure S5. Higher body mass index (BMI) correlation with oxylipin concentrations. The analysis is presented for the following sample with the corresponding indices: underweight < 18.5 (*n* [illegible]), normal weight 18.5-24.9 (*n* [illegible]8), overweight 25-29.9 (*n* [illegible] 50) and obese > 30 (*n* [illegible]5). **(A)** Volcano plot indicating significantly changed compounds. The X-axis indicates a log2 fold change of BMI index of breast cancer patients. Y-axis indicates −log10 p-values (adjusted). The cut-off for p-values is indicated based on Benjamini-Hochberg correction. Compounds that changed insignificantly are indicated in gray, compounds whose means changed in BC (relative to HCs) more than twofold or less than twofold but insignificantly are indicated in green. **(B)** The partial least square discriminant analysis (PLS-DA) model discriminating against 2 groups of BC patients (normal and high BMI index). The explained variance of each component is indicated in brackets on the corresponding axis.

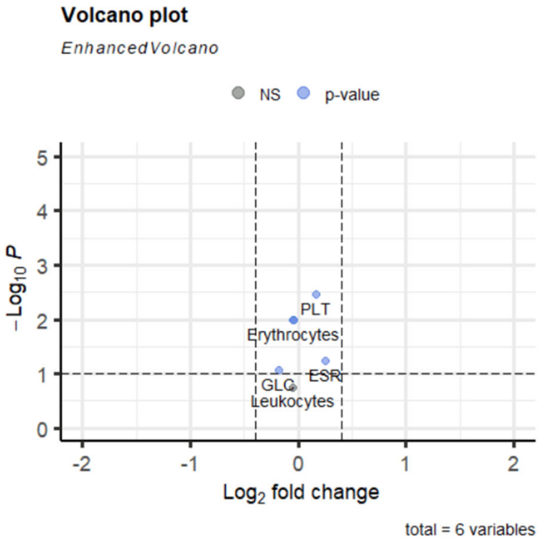

	Row.names	log2.donor_mean.BRCA_mean.	var	p.value
12	PLT	0.22000053	PLT	0.0002516768
6	HCT	-0.05660816	HCT	0.0043072301
3	Erythrocytes	-0.04074440	Erythrocytes	0.0651317657
5	GLC	-0.09440972	GLC	0.0651317657
7	HGB	-0.04355185	HGB	0.0651317657
11	NE	-0.15778081	NE	0.0651317657
9	LYM	0.22949816	LYM	0.1880532961
2	EOS	0.24188860	EOS	0.3776142472
4	ESR	0.23760483	ESR	0.3776142472
10	MONO	0.05583628	MONO	0.5090171169
1	BASO	-0.25403761	BASO	0.5268829242
8	Leukocytes	-0.03003829	Leukocytes	0.6669224054

Figure S6. Blood biochemical markers correlation with oxylipin concentrations. [illegible]or BC patients (*n* = 71) and HC donors (*n* = 126), blood test data were obtained for various biochemical parameters: leukocytes, monocytes (MONO), lymphocytes (LYM), platelets (PLT), neutrophils (NE), hemoglobin (HGB), hematocrit (HCT), glucose (GLC), erythrocyte sedimentation rate (ESR), erythrocytes, eosinophils (EOS) and basophils (BASO). Volcano plot indicating significantly changed compounds.

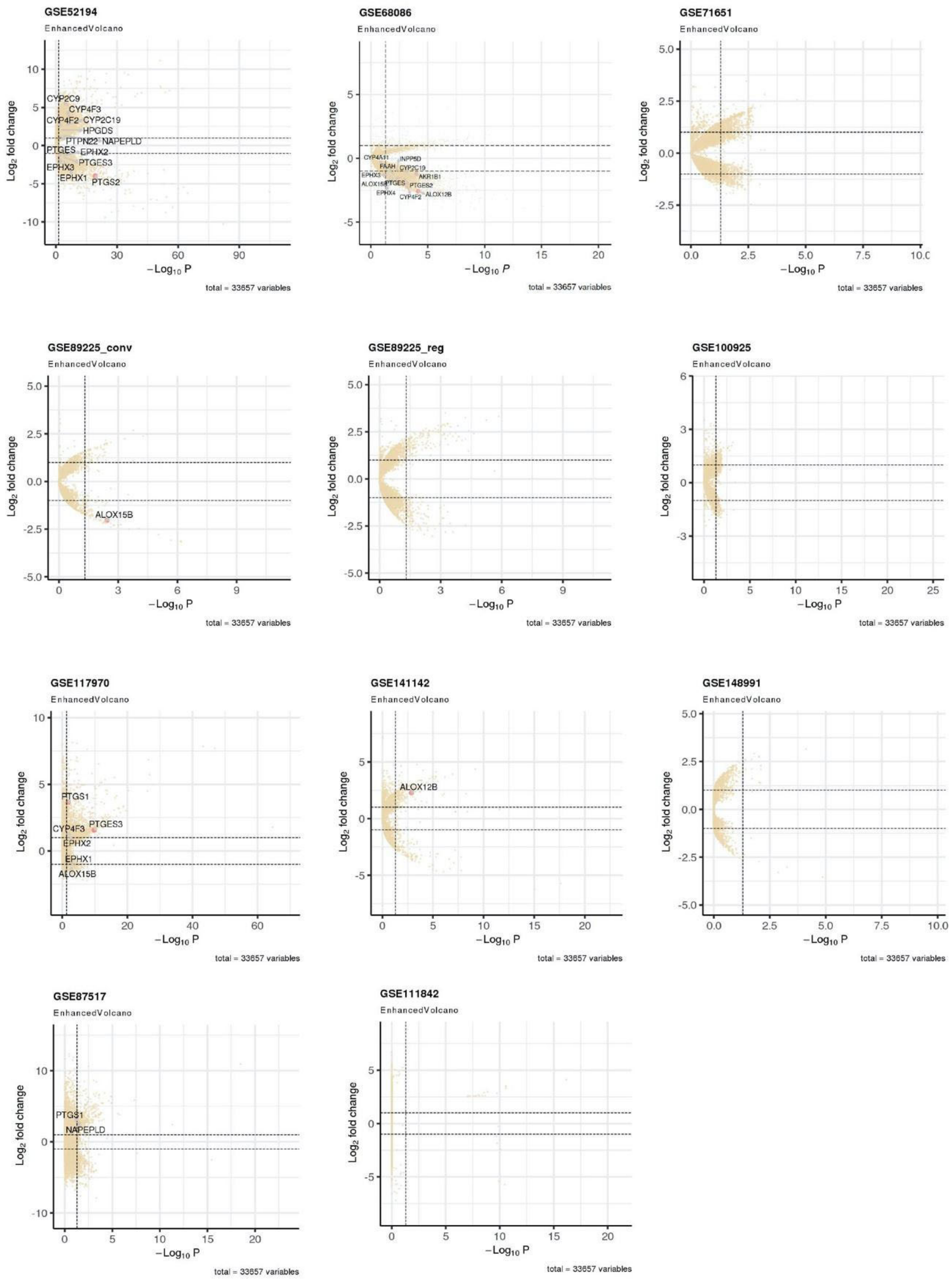

Figure S7. Volcano plots indicating significantly changed genes. The X-axis indicates −log10 *p*-values (adjusted). Y-axis indicates a log2 fold change of expression in breast cancer patients. Dashed lines show the selected threshold values for determining differential expression. Big firebrick dots correspond to differentially expressed target genes, small wheat dots correspond to others.

Table S1. UPLC-MS/MS parameters of the identified lipids.

Compound name	CAS Number	Retention time (min)	Target ion (m/z)		+/-	Matching internal standard
TXB2	54397-85-2	9,465	369,2	195,1	(−)	TXB2-d4
LXA5	110657-98-2	10,47	349,2	115,1	(−)	PGE2-d4
PGE2	363-24-6	10,7	351,2	271,2	(−)	PGE2-d4
13,14-dihydro-15-keto-PGF2a	27376-76-7	11,72	353,2	113,1	(−)	PGD2-d4
Resolvin D1	872993-05-0	11,96	375,2	141,1	(−)	PGD2-d4
PGA2	13345-50-1	13,02	333,2	271,2	(−)	PGD2-d4
17,18-DiHETE	116477-53-3	13,94	335,2	247,2	(−)	LTB4-d4
12,13-DiHOME	263399-35-5	14,46	313,2	183,1	(−)	LTB4-d4
9,10-DiHOME	263399-34-4	14,63	313,2	201,2	(−)	LTB4-d4
12-keto-LTB4	136696-10-1	14,74	333,2	179,1	(−)	LTB4-d4
14,15-DHET	77667-09-5	15,04	337,2	207,2	(−)	LTB4-d4
11,12-DHET	192461-95-3	15,36	337,2	167,1	(−)	LTB4-d4
8,9-DHET	192461-96-4	15,54	337,2	127,1	(−)	LTB4-d4
9-HOTrE	89886-42-0	15,66	293,2	171,1	(−)	LTB4-d4
20-carboxy-AA	79551-84-1	15,67	333,2	297,2	(−)	LTB4-d4
13-HODE	73804-64-5	16,6	295,2	195,1	(−)	15-HETE-d8
9-HODE	98524-19-7	16,6	295,2	171,1	(−)	15-HETE-d8
20-HDoHE	90906-41-5	16,77	343,2	241,2	(−)	15-HETE-d8
15-HETE	73836-87-0	16,93	319,2	219,2	(−)	15-HETE-d8
13-KODE	54739-30-9	17	293,2	113,1	(−)	15-HETE-d8
16-HDoHE	90780-51-1	17,05	343,2	233,2	(−)	12-HETE-d8
9-KODE	54232-59-6	17,12	293,2	185,1	(−)	12-HETE-d8
11-HETE	73804-65-6	17,125	319,2	167,1	(−)	12-HETE-d8
12-HETE	54397-83-0	17,28	319,2	179,1	(−)	12-HETE-d8
11-HDoHE	87018-59-5	17,31	343,2	149,1	(−)	5-HETE-d8
5-HETE	70608-72-9	17,38	319,2	115,1	(−)	5-HETE-d8
15-HETrE	92693-02-2	17,44	321,2	221,2	(−)	5-HETE-d8
12,13-EpOME		17,78	295,2	195,2	(−)	5-HETE-d8
9,10-EpOME		17,85	295,2	171,1	(−)	5-HETE-d8
AEA	94421-68-8	18,21	348,2	62,1	(+)	OEA-d4
OEA	111-58-0	19,14	326,2	62,1	(+)	OEA-d4
EPA	10417-94-4	19,415	301,2	257,2	(−)	EPA-d5
DHA	6217-54-5	20,16	327,2	283,2	(−)	DHA-d5
AA	506-32-1	20,32	303,2	303,2	(−)	AA-d8

Table S2. Oxylipins changed in BC compared with HC patients, human enzymes and genes involved in lipid metabolism.

Metabolite	Enzymes and *genes* involved in biosynthesis	Enzymes and *genes* involved in degradation
AA		
5-HETE	5-LOX (*ALOX5)*	5-HEDH
15-HETrE	15-LOX-2 (*ALOX15B)*	
AEA	NAPE-PLD (*NAPEPLD)*, α/β-hydrolase domain 4 (*ABDH4)*, GDE1 (*GDE1*), PTPN22 (*PTPN22*), INPP5D (*INPP5D)*	FAAH (*FAAH)*, COX-2 (*PTGS2)*, 12-LOX (*ALOX12*), 15-LOX (*ALOX15)*
11-HDoHE	12-LOX (*ALOX12*)	
9,10-EpOME	CYP2J (*CYP2J2)*, CYP2C (*CYP2C18, CYP2C19, CYP2C8, CYP2C9)*	mEH (*EPHX1)*, sEH (*EPHX2)*, EH3 (*EPHX3)*, EH4 (*EPHX4)*
12,13-EpOME	CYP2J (*CYP2J2)*, CYP2C (*CYP2C18, CYP2C19, CYP2C8, CYP2C9)*	mEH (*EPHX1)*, sEH (*EPHX2)*, EH3 (*EPHX3)*, EH4 (*EPHX4)*
PGA2+PGJ2	PGDS (*HPGDS)*, COX-1 (*PTGS1*), COX-2 (*PTGS2)*, n.e.	
9-HODE	5-LOX (*ALOX5)*	
12-HETE	12-LOX (*ALOX12)*, 15-LOX (*ALOX15*) (min)	12-HEDH
11-HETE	15-LOX-2 (*ALOX15B)*, COX-1 (*PTGS1*), COX-2 (*PTGS2)*	
LXA5	5-LOX (*ALOX5)*	
20-carboxy-AA	CYP4A11 (*CYP4A11)*, Leukotriene-B(4) omega-hydroxylase 1 (*CYP4F2)*, Leukotriene-B(4) omega-hydroxylase 2 (*CYP4F3B)*, ADH (*ADH)*	
13,14-dihydro-15-keto-PGF2a (PGFM)	15-PGDH (*HPGD)*, AKR1C3 (*AKR1C3)*, AKR1B1 (*AKR1B1)*, CBR1 (*CBR1)*	
PGE2	COX-1 (*PTGS1)*, COX-2 (*PTGS2)*, mPGES-1 (*PTGES)*, mPGES-2 (*PTGES2)*, cPGES (*PTGES3*)	15-PGDH (*HPGD)*, CBR1 (*CBR1)*
Resolvin-D1	15-LOX (*ALOX15)*, 5-LOX (*ALOX5)*	
16-HDoHE	n.e.	
20-HDoHE	n.e.	

Red - increased metabolites, Blue - decreased metabolites.

Abbreviation: Metabolites, AA - Arachidonic acid, HETE - hydroxyeicosatetraenoic acids, HETrE - hydroxyeicosatrienoic acids, AEA - anandamide, HDoHE - hydroxydocosahexaenoic acids, EpOME - epoxyoctadecamonoenoic acids, PGA2 - prostaglandin A2, PGJ2 - prostaglandin J2, HODE - hydroxyoctadecadienoic acids, LXA5 - Lipoxin A5, 20-carboxy-AA - 20-carboxy arachidonic acid, 13,14-dihydro-15-keto-PGF2a - 13,14-dihydro-15-keto Prostaglandin F2α, PGE2 - prostaglandin E2. **Proteins,** LOX - lipoxygenase, 5-HEDH - 5-Hydroxyeicosanoid dehydrogenase, NAPE-PLD - NAPE-specific phospholipase D, ABDH4 - α/β-hydrolase domain 4, GDE1 - glycerophosphodiester phosphodiesterase 1, PTPN22 - Protein Tyrosine Phosphatase Non-Receptor Type 22, INPP5D - Inositol Polyphosphate-5-Phosphatase D, FAAH - Fatty acid amide hydrolase, COX-2 - cyclooxygenase-2, PTGS2 - prostaglandin-endoperoxide synthase 2, ALOX - arachidonate -lipoxygenase, CYP2J2 - cytochrome P450 2J2, CYP2C - cytochrome P450 2C subfamily, mEH - microsomal epoxide hydrolase, sEH - soluble epoxide hydrolase, EPHX - epoxide hydrolase, EH3 - epoxide hydrolase 3, EH3 - epoxide hydrolase 4, mPGES-1 - microsomal prostaglandin E synthase-1, PGDS - Prostaglandin-D synthase, CYP4A11 - Cytochrome P450 4A11, CYP4F2 - Cytochrome P450 4F2, CYP4F3B - Cytochrome P450 F3B, ADH - alcohol dehydrogenase, 15-PGDH - 15-hydroxyprostaglandin dehydrogenase, AKR1C3 - aldo-keto reductase family 1 member C3, AKR1B1 - aldo-keto reductase family 1, member B1, CBR1- carbonyl reductase 1, n.e - non-enzymatically.

Table S3. Datasets involved in the analysis of differential gene expression.

Considered datasets	Number of samples for analysis*	Healthy group	Sick group
**Tissue datasets**			
GSE141142 (*n* = 24)	14	tissue from healthy people	tissue from BC patients
GSE52194 (*n* = 20)	20	tissue from healthy people	tissue from BC patients
GSE100925 (*n* = 103)	58	tissue from healthy people	tissue from BC patients
GSE71651 (*n* = 33)	18	tissue from healthy people	tissue from BC patients
GSE89225 (*n* = 54)	15	conventional CD4 T-cells from healthy people	conventional CD4 T-cells from BC patients
	19	regulatory T-cells from healthy people	regulatory T-cells from BC patients
**Blood datasets**			
GSE68086 (*n* = 285)	94	blood platelets from healthy people	blood platelets from BC patients
GSE117970 (*n* = 116)	79	monocytes from healthy people	monocytes from BC patients
GSE87517 (*n* = 41)	30	leukocytes (CD45+CD3+ T-cells) from healthy people	leukocytes from BC patients
GSE111842 (*n* = 34)	22	peripheral blood from healthy people	circulating tumor cells sorted from peripheral blood from BC patients
GSE148991 (*n* = 11)	11	plasma and buffy coat from healthy people	circulating tumor cells from metastatic BC patients

* the number of analyzed biological samples.

Table S4. Changes in PUFA and human oxylipins in different types of cancer.

	**Our data (*n* = 169)**	**Breast cancer, [1] (*n* = 20)**	**Ovarian cancer, [2] (*n* = 157)**	**Lung cancer, [3] (*n* = 37)**	**Prostate cancer, [4] (*n* = 19)**	**Colorectal cancer, [5] (*n* = 25)**
AA	↑	NA	NA	↑	↓	NA
PGE2 (AA)	↑	NA	-	NA	NA	-
12-keto-LTB4 (AA)	-	NA	NA	NA	NA	↓
15-HETE (AA)	-	-	NA	↑	↑	-
11-HETE (AA)	↑	-	-	↑	↑	-
12-HETE (AA)	↑	-	-	↑	-	-
5-HETE (AA)	↓	NA	-	↑	↑	-
12,13-EpOME (LA)	↓	NA	-	NA	NA	NA
9,10-EpOME (LA)	↓	NA	-	NA	NA	NA
13-HODE (LA)	-	↑	↑	-	NA	↓
9-HODE (LA)	↑	↑	↑	-	NA	↓
12,13-DiHOME (LA)	-	NA	↑	NA	NA	-
9-HOTrE (α-LA)	-	↑	NA	NA	NA	NA

NA = not analyzed.

References

1. Azbukina, N.V.; Lopachev, A.V.; Chistyakov, D.V.; Goriainov, S.V.; Astakhova, A.A.; Poleshuk, V.V.; Kazanskaya, R.B.; Fedorova, T.N.; Sergeeva, M.G. Oxylipin profiles in plasma of patients with wilson's disease. *Metabolites* **2020**, *10*, 222. https://doi.org/10.3390/metabo10060222.
2. Gavrish, G.E.; Chistyakov, D.V.; Sergeeva, M.G. ARGEOS: A new bioinformatic tool for detailed systematics search in GEO and arrayexpress. *Biology* **2021**, *10*, 1026. https://doi.org/10.3390/BIOLOGY10101026.
3. Piñero, J.; Bravo, Á.; Queralt-Rosinach, N.; Gutiérrez-Sacristán, A.; Deu-Pons, J.; Centeno, E.; García-García, J.; Sanz, F.; Furlong, L.I. DisGeNET: A comprehensive platform integrating information on human disease-associated genes and variants. *Nucleic Acids Res.* **2017**, *45*, D833–D839. https://doi.org/10.1093/NAR/GKW943.
4. Wu, C.C.; Gupta, T.; Garcia, V.; Ding, Y.; Schwartzman, M.L. 20-HETE and blood pressure regulation: Clinical implications. *Cardiol. Rev.* **2014**, *22*, 1–12. https://doi.org/10.1097/CRD.0B013E3182961659.
5. Guryleva, M.V.; Chistyakov, D.V.; Lopachev, A.V.; Goriainov, S.V.; Astakhova, A.A.; Timoshina, Y.A.; Khutorova, A.V.; Fedorova, T.N.; Sergeeva, M.G. Modulation of the Primary Astrocyte-Enriched Cultures' Oxylipin Profiles Reduces Neurotoxicity. *Metabolites* **2021**, *11*, 498. https://doi.org/10.3390/METABO11080498.
